# Supplementary material for: WordCommentsAnalyzer: A windows software tool for qualitative research
Source: F1000Res. 2018 Sep 4;7:536. Originally published 2018 May 3. [Version 2] doi: 10.12688/f1000research.14819.2 (PMC6137411; doi:10.12688/f1000research.14819.2)
Supplement: Supplementary file 3 [file f1000research-7-17614-s0002.tgz › b2a79496-4ada-47c4-9682-a99ce867836d.zip!/Eight Iranian Health Journals/Archives of Rehabilitation_19.docx]

© Archives of Rehabilitation. This work is licensed under CC BY-NC 4.0 (https://creativecommons.org/licenses/by-nc/4.0/).

Volume & Issue: دوره 14 -

Title: میزان تنظیم هیجان در مصرف‌کنندگان مت‌آمفتامین با سابقه رفتار‌‌های مخاطره‌آمیز و مصرف‌کنندگان بدون سابقه رفتار‌‌های مخاطره‌آمیز

http://rehabilitationj.uswr.ac.ir/article-1-1452-fa.html

هدف: افراد برای فرار از فشار‌‌های روانی، ممکن است به انکار وجود آنها به هر وسیله ممکن بپردازند. در این صورت با آگاهی و اصرار، به انجام رفتار‌‌های پرخطر (از قبیل مصرف مواد و تزریق آن در افراد معتاد) گرایش پیدا می‌کنند. عدم توانایی در حل مشکلات و درگیری‌‌‌های هیجانی، منجر به بروز رفتار‌‌های پرخطر در افراد معتاد می‌شود. هدف مطالعه حاضر بررسی ارتباط سابقه رفتار‌‌های مخاطره‌آمیز (سابقهٔ زندان و تزریق مواد)، با میزان تنظیم هیجان در مصرف‌کننده‌‌‌های مت‌آمفتامین مراجعه‌کننده به مراکز درمانی بود. روش‌ بررسی:‌ در یک مطالعه مورد-شاهدی تعداد ۶۰نفر از مصرف‌کننده‌های مت‌آمفتامین مراجعه‌کننده به مراکز درمانی دردسترس (کلینیک درمان با داروهای آگونیست، کمپ و گروه‌های خودیاری و انجمن معتادان گمنام) با روش نمونه‌گیری هدفمند انتخاب شدند. اطلاعات جمعیت‌شناختی و سابقه رفتار‌‌های مخاطره‌آمیز در این گروه اخذ گردید. برای به‌دست آوردن نمره تنظیم هیجانی از پرسشنامه شناختی تنظیم هیجان (CERQ) استفاده شد. داده‌‌‌های پژوهش با استفاده از آزمون آنالیز واریانس چندمتغیره تحلیل شد. یافته‌ها: نتایج این پژوهش نشان داد که تفاوت دو گروه دارای رفتار مخاطره‌آمیز و بدون رفتار مخاطره‌آمیز در هیچ‌یک از خرده‌مقیاس‌‌‌های پرسشنامه شناختی تنظیم هیجان معنادار نمی‌باشد. نتیجه‌گیری: ارتباط معناداری بین توانایی تنظیم هیجانی و خرده‌مقیاس‌های آن با ارتکاب رفتارهای مخاطره‌آمیز زندان و تزریق مواد در مصرف‌کنندگان مت‌آمفتامین دیده نمی‌شود.

Title: بررسی رابطه افسردگی، اضطراب، و استرس با ابراز پرخاشگری در افراد وابسته به مصرف مواد مخدر

http://rehabilitationj.uswr.ac.ir/article-1-1466-fa.html

هدف: هدف پژوهش حاضر تعیین رابطه افسردگی، اضطراب، استرس، با پرخاشگری در افراد وابسته به مصرف مواد مخدر بود. روش‌ بررسی: مطالعه تحلیلی حاضر از نوع تعیین همبستگی است و نمونه پژوهش، ۱۱۰ نفر از افراد وابسته به مصرف مواد مخدر در سال ۱۳۹۱ بودند که به شیوه نمونه‌گیری در دسترس انتخاب شدند. ابزار این پژوهش عبارت بود از: مقیاس افسردگی، اضطراب، استرس داس و پرسشنامه پرخاشگری باس و پری. تحلیل داده‌ها از طریق ضریب همبستگی پیرسون و رگرسیون چندمتغیری انجام شد. یافته‌ها: نتایج ضریب همبستگی پیرسون رابطه معناداری را بین افسردگی، اضطراب، و استرس با پرخاشگری نشان داد (افسردگی با پرخاشگری ۰/۸۲۸=r، اضطراب با پرخاشگری ۰/۷۹۳=r، و استرس با پرخاشگری (۰/۷۷۵=r؛ ۰/۰۰۰۵>P). همچنین، افسردگی با سطح معناداری (P<0/0005) و استرس با سطح معناداری (P>0.05) پیش‌بینی‌کنندگی معناداری را با پرخاشگری نشان دادند، ولی اضطراب پیش‌بینی‌کننده معناداری نبود (P<0.05). نتیجه‌گیری: نتایج پژوهش نشان داد که بین افسردگی، اضطراب، استرس با پرخاشگری در افراد وابسته به مصرف مواد مخدر رابطه وجود دارد. این مطالعه پیشنهاد می‌کند مراکز درمان اعتیاد و کلینیک‌های روانشناختی، هم‌ابتلایی مصرف مواد مخدر با دیگر اختلالات روانی را در افراد وابسته به مواد در نظر داشته باشند و نسبت به درمان آنها نیز اقدام شود.

Title: نقش ادراک خطر و خودتأملی در پیش‌بینی انگیزش درمان افراد وابسته به مواد

http://rehabilitationj.uswr.ac.ir/article-1-1460-fa.html

هدف: انگیزش درمان عنصری کلیدی در درمان و بهبودی از اختلالات مصرف مواد می‌باشد. پژوهش حاضر با هدف تعیین نقش ادراک خطر و خودتأملی در پیش‌بینی انگیزش درمان افراد وابسته به مواد انجام گرفت. روش بررسی: این پژوهش پیمایشی از طریق تعیین همبستگی انجام شد. کلیه افراد دارای وابستگی به مواد که در نیمه اول سال ۱۳۹۲ به مراکز ترک اعتیاد شهر اردبیل مراجعه کرده و تحت درمان بودند جامعه آماری این پژوهش را تشکیل دادند. تعداد ۱۴۰ نفر از این افراد به روش نمونه‌گیری تصادفی خوشه‌ای انتخاب شده و به پرسش‌نامه‌های ادراک خطر، خودتأملی و انگیزش درمان پاسخ دادند. داده‌های جمع‌آوری شده نیز با استفاده از آزمونهای همبستگی پیرسون و تحلیل رگرسیون چند گانه تحلیل شد. یافته‌ها: نتایج ضرایب همبستگی نشان داد که انگیزش درمان با ادراک خطر مصرف داروی محرک (۰/۲۶=r؛ ۰/۰۰۳>P)، خطر رفتار پرخاشگری (۰/۴۷=r؛ ۰/۰۰۱>P)، خطر فعالیت‌های ورزشی (۰/۲۱=r؛ ۰/۰۱۵>P)، خطر شکست تحصیلی و شغلی (۰/۶۸=r؛ ۰/۰۰۱>P)، خطر رفتار جنسی (۰/۳۱=r؛ ۰/۰۰۱>P) و خطر می‌بارگی (۰/۴۳=r؛ ۰/۰۰۱>P) رابطه مثبت دارد. همچنین انگیزش درمان با نمره کلی خودتأملی (۰/۲۴=r؛ ۰/۰۰۶>P) و نیاز به خودتأملی (۰/۴۲=r؛ ۰/۰۰۱>P) رابطه مثبت داشت. نتایج تحلیل رگرسیون نیز نشان داد ۵۰ درصد از واریانس انگیزش درمان به وسیله ادراک خطر و ۱۹ درصد آن به وسیله خودتأملی تبیین می‌شود. نتیجه‌گیری: نتایج این پژوهش نشان می‌دهد که ادراک بالای خطرات رفتاری ناشی از مصرف دارو و توانایی بالای خود تأملی می‌توانند به صورت معنی‌داری انگیزش درمان و آمادگی افراد وابسته به مواد برای تغییر را پیش‌بینی کنند.

Title: بررسی وضعیت و روند سوءمصرف و وابستگی به مواد در زنان ایرانی

http://rehabilitationj.uswr.ac.ir/article-1-1456-fa.html

هدف: هدف مطالعه بررسی وضعیت و روند سوءمصرف مواد در زنان ایرانی مبتلا به اختلال وابستگی به مواد بود. روش‌های بررسی: مطالعه کیفی و توصیفی است. از بین زنان مصرف‌کننده مواد که جهت درمان و بازتوانی به کلینیک‌های دولتی، خصوصی و خیریه مراجعه نموده‌اند به‌صورت تصادفی نمونه‌ای ۳۰نفره، که حداقل ۲ سال واجد معیار تشخیصی وابستگی به مواد بودند، انتخاب شدند و مورد مصاحبه قرار گرفتند. یافته‌ها: محدوده سنی ۲۶تا۳۰ سال اوج مصرف مواد در زنان است که ماده مصرفی طی یکماه قبلی از مراجعه برای درمان، کراک ٪۶۰، شیشه ٪۴۶/۶، میزان مصرف برحسب گرم روزانه ۱تا ۱/۵ گرم است. روش مصرف ٪۸۰ کشیدنی، ٪۶/۷ استنشاقی و ٪۱۳/۳ تزریق بود. ٪۷۳/۳ مواد مصرفی خود را تغییر داده‌اند. علت تغییر نوع ماده مصرفی کسب لذت بیشتر و شایع‌ترین علت شروع مصرف مواد، اعتیاد همسر و بیشترین الگوی درمانی قبلی، پزشکی و طبی بود. نتیجه‌گیری: روند سو‌ءمصرف مواد در زنان بسیارخطرناکتر و متفاوت از مردان است. زنان در طول دوره اعتیاد حمایت کمتری از نظر مالی و عاطفی از همسر و اعضای خانواده و اجتماع دریافت می‌کنند. تغییر الگوی سوءمصرف مواد در زنان نسبت به سالهای گذشته و افزایش شیوع سوءمصرف مواد صنعتی و کاهش سن ابتلا در زنان از نتایج این پژوهش است.

Title: مطالعه اثربخشی برنامه مصون‌سازی کودکان پیش‌دبستانی در برابر مواد اعتیادآور در مراکز پیش‌دبستانی شهر اصفهان

http://rehabilitationj.uswr.ac.ir/article-1-1362-fa.html

هدف: پژوهش حاضر با هدف بررسی اثربخشی برنامه مصون‌سازی کودکان پیش‌دبستانی در برابر مواد اعتیادآور در مراکز پیش‌دبستانی شهر اصفهان اجرا گردید. روش بررسی: به‌منظور دست یابی به هدف فوق، ۶۰ کودک از مهدکودک‌های شهر اصفهان به شیوه نمونه‌گیری در دسترس انتخاب شدند و با طرح پژوهشی شبه‌آزمایشی از نوع پیش‌آزمون-پس‌آزمون در دو گروه آزمایش، کنترل گمارده شدند. مداخلات مربوط به برنامه مصون‌سازی (بسته آموزشی کادو) بر روی گروه آزمایش انجام گرفت. آزمون مورد استفاده در این پژوهش چک‌لیست محقق‌ساخته ارزیابی دانش، عواطف و رفتار نسبت به مواد اعتیادآور درکودکان پیش‌دبستانی بود. داده‌ها با استفاده از روش آماری تحلیل کوواریانس مورد تجزیه و تحلیل قرار گرفت. یافته‌ها: پس از مداخله به‌طور معناداری دانش کودکان افزایش یافت؛ نگرش و عواطف‌شان به مواد اعتیادآور و فعالیتهای مخاطره‌آمیز منفی شد و نسبت به آن رفتار ابرازی و اجتنابی داشتند. نتیجه‌گیری: مطابق یافته‌های فوق بسته آموزشی برنامه مصون‌سازی بر کودکان پیش‌دبستانی در برابر مواد اعتیاد‌آور اثربخش است.

Title: پیش‌بینی مصرف مواد براساس ویژگی‌های شخصیتی در دانشجویان

http://rehabilitationj.uswr.ac.ir/article-1-1423-fa.html

هدف: مطالعه حاضر با هدف بررسی ارتباط ویژگیهای شخصیتی با مصرف مواد در دانشجویان دانشگاه‌های تهران صورت گرفت. روش بررسی: پژوهش حاضر در قالب طرح‌های همبستگی و از نوع مطالعات پیش‌بینی می‌باشد. نمونه مورد پژوهش دربرگیرنده ۹۱۴ دانشجو با میانگین سنی ۲۳٫۱۳ سال است که در یکی از دانشگاه‌های شهر تهران در مقاطع کارشناسی یا کارشناسی ارشد مشغول به تحصیل بوده و با استفاده از روش نمونه‌گیری خوشه‌ای انتخاب شده‌اند. به‌منظور جمع‌آوری اطلاعات موردنیاز از پرسشنامه‌های پنج عامل بزرگ شخصیت (گولدبرگ، ۱۹۹۹) و پرسشنامه رفتارهای پرخطر بررسی ملی رفتارهای پرخطر دانشجویان امریکا(NCHRBS) نسخه ۲۰۰۷ استفاده شد. متغیر ملاک یا وابسته عبارت است از "مصرف مواد" و عوامل شخصیتی، متغیرهای پیش‌بین یا مستقل را تشکیل می‌دهند. داده‌ها با استفاده از روش رگرسیون چندگانه تحلیل شد. یافته‌ها: مصرف مواد با عوامل شخصیتی "توافق" و "گشودگی تجربی" همبستگی مثبت و با عوامل شخصیتی "برون‌گرایی"، "وظیفه‌شناسی" و "پایداری هیجانی" همبستگی منفی معنا‌دار نشان داد. در پیش‌بینی مصرف مواد متغیرهای "وظیفه‌شناسی"، "توافق"، "پایداری هیجانی" و "گشودگی تجربی" به ترتیب از بیشترین نقش برخوردار هستند و مدل رگرسیون ارائه شده به‌طور کلی می‌تواند ۴۵ درصد از کل واریانس نمره مصرف مواد را تبیین نماید. نتیجه‌گیری: عوامل شخصیتی دارای جنبه‌های تعیین‌کننده بر رفتار مصرف مواد است. همچنین با امکان پیش‌بینی مصرف مواد از روی صفات شخصیتی، می‌توان افراد درمعرض خطر را شناسایی و مداخلات پیشگیرانه را بر آنها متمرکز کرد.

Title: مقایسه شاخص‌‌های کیفیت زندگی در مراقبان بیمار اسکیزوفرنیک با و بدون وابستگی به مواد

http://rehabilitationj.uswr.ac.ir/article-1-1455-fa.html

هدف: این مطالعه با هدف بررسی و مقایسه شاخص‌‌های کیفیت زندگی در مراقبان بیمار اسکیزوفرنیک (با وابستگی به مواد و بدون وابستگی بدون مواد) در مرکز روانپزشکی رازی انجام شده است. روش بررسی: در این مطالعه تحلیلی حجم نمونه‌ای ۳۰نفره از دو گروه مراقبان بیماران اسکیزوفرنیک مبتلا و غیرمبتلا به سوءمصرف مواد به‌طور ساده و دردسترس از بیماران مرکز روانپزشکی رازی انتخاب شدند. کیفیت زندگی آنها با پرسشنامه کیفیت زندگی WHO سنجیده و نتایج با آزمون تی مستقل تحلیل شد. یافته‌ها: نتایج کلی پژوهش حاضر نشان داد که کیفیت زندگی در تمامی ابعاد آن در مراقب بیمار اسکیزوفرنیک غیروابسته به مواد و مراقب بیمار اسکیزوفرنیک وابسته به مواد مشابه می‌باشد. در دو گروه مراقبان، به دلیل درگیری‌‌های مکرر جسمی و روانی با بیمار شاخص سلامت جسمی پایین بوده، استیگمای ناشی از بیماری روانی باعث افت شاخص وضعیت اجتماعی و نیز شاخص مذهبی شده و در عوض میانگین سایر شاخص‌‌های کیفیت زندگی (وضعیت محیطی، وضعیت استقلال) به دلیل تلاش‌‌های مکرر اجتماعی و محیطی خانواده‌‌ها در زمینه کمک‌خواهی برای حل مسائل‌شان بالا رفته است. نتیجه‌گیری: بنظر می‌رسد اعتیاد به مواد در بیماران اسکیزوفرنیک تأثیری بر کیفیت زندگی مراقبان آنها نداشته باشد.

Title: مقایسه ویژگی‌های شخصیتی بر اساس ابعاد سرشت و منش در سوءمصرف‌کنندگان مواد مخدر و محرک

http://rehabilitationj.uswr.ac.ir/article-1-1446-fa.html

هدف: بررسی و مقایسه ویژگی‌های شخصیتی بر اساس ابعاد سرشت و منش در معتادان مصرف‌کنندهٔ مواد افیونی و محرک با کنترل سن هدف این پژوهش بوده است. روش بررسی: در این پژوهش مقطعی‌مقایسه‌ای تعداد ۱۱۵نفر (۹۱نفر مصرف‌کننده مواد افیونی و ۲۴نفر مصرف‌کننده مواد محرک) از افراد مراجعه‌کننده به مراکز درمان سوءمصرف مواد (کلینیک‌های درمان با داروهای آگونیست)، کمپ‌ها و گروه‌های خودیاری و انجمن معتادان گمنام شهر یاسوج، با روش نمونه‌گیری ساده و در دسترس انتخاب شدند. ابزار جمع‌آوری داده‌ها پرسشنامه شخصیتی سرشت و منش (TCI) بود. داده‌ها با استفاده از آزمون‌های لوین، آنووا و ضریب همبستگی پیرسون تجزیه‌وتحلیل شد. یافته‌ها: نتایج نشان داد که در ابعاد نوجویی، آسیب‌پرهیزی، پشتکار، همکاری، خودراهبری، خودفراروی در مصرف‌کنندگان مواد افیونی و محرک تفاوت معنادار وجود دارد و در تمام مقیاس‌های فوق میانگین نمره درمواد محرک بالاتر از مواد افیونی بوده است. از نظر سن اختلاف معناداری بین دو گروه در این مؤلفه‌ها وجود نداشت. نتیجه‌گیری: بر اساس میانگین نمرات سرشت و منش، وجود و بروز ویژگی‌های شخصیتی خاص که نشانگر اختلالات شخصیتی مرتبط هستند، در بین مصرف‌کنندگان مواد قابل تشخیص و پیش‌بینی است. این موضوع در پیشگیری و درمان بیماری اعتیاد حائز اهمیت است.

Title: اثربخشی درمان شناختی-‌رفتاری گروهی مدیریت خشم بر کاهش همسرآزاری و افزایش رضایت زناشویی افراد مبتلا به سوء‌مصرف مواد

http://rehabilitationj.uswr.ac.ir/article-1-1465-fa.html

هدف: هدف پژوهش حاضر تعیین اثربخشی درمان شناختی-رفتاری مدیریت خشم بر کاهش همسرآزاری و افزایش رضایت زناشویی افراد مبتلا به سوءمصرف مواد بود. روش بررسی: طرح این پژوهش شبه‌آزمایشی از نوع پیش‌آزمون و پس‌آزمون همراه با گروه کنترل بود. جامعه آماری کلیه افراد مبتلا به سوءمصرف مواد مخدر (مرد) در تهران در سال ۱۳۹۲بودند که برای درمان به مرکز ترک اعتیاد رهپویان رهایی مراجعه کرده بودند. نمونه این پژوهش عبارت بود از ۱۲نفر از افراد مبتلا به سوءمصرف مواد مخدر که میزان بالایی از همسرآزاری را نشان داده بودند و همسران آنان که به‌صورت تصادفی انتخاب شده و نیمی از آنان در گروه آزمایش و نیمی دیگر در گروه کنترل جایگزین شدند. ابزار پژوهش عبارت بود از: پرسشنامه همسرآزاری قهاری و همکاران و پرسشنامه رضامندی زناشویی اینریچ. داده‌ها با روش آماری تی اختلافی و تحلیل کوواریانس مورد تجزیه و تحلیل قرار گرفتند. یافته‌ها: نتایج پس‌آزمون نشان داد درمان شناختی-رفتاری گروهی مدیریت خشم باعث کاهش معنادار میزان همسرآزاری (P<0/01) و افزایش رضامندی زناشویی (P<0/05, F:12/36) در گروه آزمایش در مقایسه با گروه کنترل شده است. نتیجه‌گیری: درمان شناختی-رفتاری گروهی مدیریت خشم در کاهش همسرآزاری و افزایش رضامندی زناشویی افراد مبتلا به سوءمصرف مواد مخدر مؤثر است.

Title: محتوای مناسب برنامۀ درسی پیشگیری از اعتیاد از دیدگاه معلمان و دانش‌آموزان

http://rehabilitationj.uswr.ac.ir/article-1-1448-fa.html

هدف: هدف این پژوهش بررسی محتوای مناسب برای برنامه درسی پیشگیری از اعتیاد از نظر دانش آموزان و معلمان است. روش بررسی: این پژوهش از نوع پیمایشی است. به روش تصادفی خوشه‌ای، ۲۱۰دبیر و ۳۷۰ دانش‌آموز از دانش‌آموزان دوره متوسطه (دختر و پسر) شهر همدان و دبیران دبیرستان‌های مربوطه انتخاب شدند. برای جمع‌آوری اطلاعات از دو پرسشنامهٔ محقق‌ساخته استفاده شد. در نهایت اطلاعات اخذشده از ۳۶۳ دانش‌آموز و ۲۰۰ دبیر بااستفاده‌از آزمون‌های آماری مجذور خی، فریدمن و تی مستقل تحلیل شد. یافته‌ها: بیشترین توافق برای محتوای مناسب درسی از نظر گروهها به ترتیب مهارت‌های «نه» گفتن به خواسته‌های نامعقول دیگران، چگونگی تأثیر دوستان در ابتلا و پیشگیری از اعتیاد و مهارتهای تصمیم‌گیری و برقراری ارتباط با دیگران است. بین سه مولفۀ علل، آسیب و پیشگیری از اعتیاد از نظر اهمیت تفاوت معناداری وجود نداشت. بین نظرات گروه‌های مختلف (معلمان و دانش‌آموزان) و جنسیت مختلف تفاوت معنادار وجود داشت. نتیجه‌گیری: براساس یافته‌ها می‌توان نتیجه گرفت که از نظر معلمان و دانش‌آموزان مطرح کردن این مباحث در کتب درسی دورۀ متوسطه مناسب است. اما برای مطرح شدن آن باید روی مباحثی که بیشترین میزان توافق بر سر آن وجود دارد تأکید شود.

Title: ارزیابی و اولویت‌بندی روش‌‌‌های درمان اعتیاد با استفاده از تکنیک تاپسیس

http://rehabilitationj.uswr.ac.ir/article-1-1463-fa.html

هدف: این مطالعه جهت ارزیابی و انتخاب مناسب‌‌ترین روش سم‌زدائی از بین چهار روش علامتی، روش داروی آگونیست (جایگزینی)، روش سم‌زدائی سریع و روش سم‌زدائی فوق سریع انجام شده است. روش‌ بررسی: سی فرد خبره چهار روش سم‌زدائی را از نظر هفت معیار ذکر شده در نظر گرفته و برای ارزیابی هر گزینه نسبت به هر معیار از مقیاس ۱ تا ۹ (مقیاس دو قطبی) استفاده کردند. پس از تشکیل ماتریس تصمیم‌گیری در یک فرآیند شش مرحل‌‌های تکنیک تاپسیس چهار روش سم‌زدائی را اولویت‌بندی کرده و مناسب‌ترین روش مشخص شد. یافته‌ها: روش سم‌زدایی با داروی آگونیست (جایگزینی) نسبت به سایر روش‌ها، همزمان به معیار‌‌های مثبت نزدیک‌تر و از معیار‌‌های منفی دورتر است(۰/۶۵۳۴۴۶ Ci). نتیجه‌گیری: مطابق یافته‌های این تحقیق، روش سم‌زدایی با داروی آگونیست (جایگزینی) مناسب‌ترین روش سم‌زدائی می‌باشد.

Title: ارزیابی خدمات پیشگیری، درمان و کاهش آسیب اعتیاد با تمرکز بر عفونت ویروس نقص ایمنی انسانی به پناه‌جویان افغانی در ایران از منظر ارائه‌دهندگان این خدمات

http://rehabilitationj.uswr.ac.ir/article-1-1472-fa.html

هدف: جمهوری اسلامی ایران در چارچوب همکاری‌های دو و چند جانبه با دفتر مبارزه با مواد و جرم سازمان ملل و کشورهای همسایه ارائه خدمات پیشگیری از ویروس نقص ایمنی به پناهجویان افغان مصرف‌کننده مواد در ایران و پاکستان و بازگشتگان به افغانستان، را در قالب یک پروژه مشترک در دستور کار قرار داده است. در این پروژه خدمات امداد رسانی اجتماع محور از طریق همتا آموزی، ترویج و توزیع کاندوم، خدمات بهداشتی اولیه شامل درمان آبسه‌های عفونی، برنامه سرنگ و سوزن، درمان جایگزین مواد افیونی، ارجاع داوطلبانه به مراکز مشاوره و تست HIV، ارجاع در موارد خدمات درمان ضد ویروسی، خدمات روانی، اجتماعی و همچنین آزمایش و درمان سل، هپاتیت ث و هپاتیت ب به پناهندگان افغانی در ایران ارائه می‌شود. روش بررسی: به‌منظور پایش و ارزیابی مرحله اول اجرایی این پروژه که در سال ۱۳۸۹ در شهرستان‌های مشهد و ورامین و در چهار مرکز گذری آغاز شد، به روشی کیفی، یک جلسه بحث گروهی متمرکز با شرکت ده نفر از کارشناسان، مجریان و مدیران اجرایی طرح در تاریخ ۲۷ دی ۱۳۹۰ در محل اداره کل درمان و حمایت‌های اجتماعی ستاد مبارزه با مواد مخدر ریاست جمهوری تشکیل گردید. مقاله حاضر نقاط قوت و ضعف و چالش‌های این برنامه را با توجه به نظرات کارشناسان، مدیران و مجریان برنامه بررسی می‌نماید. یافته‌ها: تغییرات مثبتی در جهت اهداف فوق حاصل شده است. ازجمله: افزایش آگاهی‌های پناه‌جویان، کاهش رفتارهای پرخطری مثل استفاده از سرنگ مشترک و ارتباطات جنسی لجام‌گسیخته، افزایش آزمایشات اچ‌آی‌وی و شناسایی ناقلین و ارجاع به مراکز درمانی. نتیجه‌گیری: پروژه دفتر مبارزه با مواد و جرم سازمان ملل برای ارائه خدمات پیشگیری، درمانی و کاهش آسیب عفونت نقص ایمنی انسانی به پناه‌جویان افغانی مصرف‌کننده مواد در ایران و پاکستان و بازگشتگان به افغانستان پاسخی به چالش موجود در این زمینه است.
